# Prognostic impact of FAN score in patients receiving nivolumab plus ipilimumab for metastatic renal cell carcinoma

**DOI:** 10.1038/s41598-024-63403-2

**Published:** 2024-05-29

**Authors:** Shimpei Yamashita, Shuzo Hamamoto, Junya Furukawa, Kazutoshi Fujita, Masayuki Takahashi, Makito Miyake, Noriyuki Ito, Hideto Iwamoto, Yasuo Kohjimoto, Isao Hara

**Affiliations:** 1 Department of Urology, Wakayama Medical University, 811-1 Kimiidera, Wakayama, 641-0012 Japan; 2 Department of Nephro-Urology, Nagoya City University Graduate School of Medical Sciences, Nagoya, Japan; 3 Department of Urology, Kobe University Graduate School of Medicine, Kobe, Japan; 4 Department of Urology, Kindai University Faculty of Medicine, Osakasayama, Japan; 5 Department of Urology, Tokushima University Graduate School of Biomedical Sciences, Tokushima, Japan; 6 Department of Urology, Nara Medical University, Kashihara, Nara Japan; 7 Department of Urology, Japanese Red Cross Wakayama Medical Center, Wakayama, Japan; 8 Division of Urology, Department of Surgery, School of Medicine, Faculty of Medicine, Tottori University, Yonago, Japan

**Keywords:** Oncology, Urology

## Abstract

FAN score is reportedly associated with prognostic outcomes in patients with urothelial carcinoma being treated with immune check point inhibitors. However, the prognostic impact of pre-treatment FAN score in patients with metastatic renal cell carcinoma (RCC) treated with nivolumab plus ipilimumab remains unclear. We retrospectively evaluated the association between pre-treatment FAN score and prognostic outcomes in 154 patients with metastatic RCC treated with nivolumab plus ipilimumab. The pre-treatment FAN score was ‘0’ in 56 patients (36%), ‘1’ in 60 patients (40%), ‘2’ in 37 patients (24%) and ‘3’ in one patient (1%). Progression-free survival was not significantly different between patients with different FAN scores, but second progression-free survival (PFS2), cancer-specific survival (CSS) and overall survival (OS) were significantly different. In multivariable Cox proportional hazard analyses, FAN score ≥ 2 was a significant predictor of poor PFS2 (vs. FAN score 0, HR: 2.43, 95% CI 1.21–4.87, *P* = *0.01*), poor CSS (vs. FAN score 0, HR: 2.71, 95% CI 1.13–6.47,* P* = *0.02*) and poor OS (vs. FAN score 0, HR: 2.42, 95% CI 1.11–5.25, *P* = *0.02*). High pre-treatment FAN score could be a significant independent predictor of poor prognosis in patients receiving nivolumab plus ipilimumab for metastatic RCC.

## Introduction

Until recently, the main treatment option for advanced renal cell carcinoma (RCC) was molecular targeted therapy represented by tyrosine kinase inhibitors. The introduction of immune checkpoint inhibitors (ICIs) has greatly altered the treatment strategy for advanced RCC in recent years^1^. Several randomized clinical trials have shown that combination immunotherapies (either a combination of two ICIs, or an ICI and a tyrosine kinase inhibitor) provide significant clinical benefits as systemic first-line therapies compared with sunitinib^2–7^. Among these combinations, nivolumab plus ipilimumab is currently the only treatment regimen to combine multiple ICIs, namely anti-programmed death-1 (PD-1) and anti-cytotoxic T lymphocyte-associated antigen 4. Nivolumab plus ipilimumab is recommended as the first-line systemic therapy for patients with International Metastatic Renal Cell Carcinoma Database Consortium (IMDC) intermediate/poor-risk disease^8^.

Extended (over 5-year) follow-up data of Checkmate 214 trial has shown that nivolumab plus ipilimumab treatment continues to provide durable clinical benefits compared with sunitinib during long-follow up duration, and with relatively long median overall survival (OS) (47 months)^9^. On the other hand, the best overall response was progressive disease in about 20% of patients, suggesting the possibility that not all patients benefit from nivolumab plus ipilimumab. Development of novel prognostic markers or classifications for patients treated with ICIs is therefore required.

Recently, FAN score, which is based on fibrosis-4 (Fib-4) index, albumin–bilirubin (ALBI) score and neutrophil–lymphocyte ratio (NLR), was reported to be associated with prognostic outcomes in patients with metastatic urothelial carcinoma treated with pembrolizumab^10^. However, no previous studies have reported the impact of pre-treatment FAN score on the prognosis of patients with metastatic RCC receiving immunotherapies including nivolumab plus ipilimumab. This multi-center retrospective study aims to investigate the association between pre-treatment FAN score and the prognosis in patients with metastatic RCC treated with nivolumab plus ipilimumab.

## Materials and methods

### Patients

We retrospectively reviewed the medical records of consecutive patients who received nivolumab plus ipilimumab treatment for metastatic RCC at eight institutions (our hospital and seven collaborating hospitals) between October 2015 and May 2022. Of 173 patients, this study included the 154 patients whose pre-treatment FAN score could be calculated.

### Data collection

Patient characteristics, including laboratory data at the time of drug administration, were retrospectively collected. The collected clinical features were age, gender, body mass index, IMDC risk classification, complete blood cell counts, serum C-reactive protein (CRP) level, serum albumin level, serum bilirubin level, serum aspartate aminotransferase (AST) level and serum alanine aminotransferase (ALT) level. We also collected tumor characteristics including prior resection of the primary site, histologic subtype, sarcomatoid features, metastatic sites and the number of metastatic organs. Fib-4 index was calculated using the following formula^11^:

$$ \left( {{\text{age}}\left[ {{\text{years}}} \right] \times {\text{AST}}\left[ {{\text{IU}}/{\text{L}}} \right]} \right)/({\text{platelet}}\;{\text{count}}\left[ {10^{9} /{\text{L}}} \right] \times \left( {{\text{ALT}}\left[ {{\text{IU}}/{\text{L}}} \right]^{0.5} } \right) $$

ALBI score was calculated using the following formula^12^:

$$ \left( {{\text{log}}10\left( {{\text{T - bil}}\left[ {{\text{mg}}/{\text{dL}}} \right] \times 17.1} \right) \times 0.66} \right) + \left( {{\text{albumin}}\left[ {{\text{g}}/{\text{dL}}} \right] \times 10 \times \left( { - 0.085} \right)} \right) $$

NLR was calculated as neutrophil count divided by lymphocyte count. The pre-treatment FAN score was defined as the total number of Fib-4 > 3.5, ALBI score ≥ 2.6 and NLR > 5.0, in accordance with the original study^10^.

The response of tumors to the treatment was evaluated by using Response Evaluation Criteria in Solid Tumors version 1.1^13^. The objective response rate (ORR) was defined as the rate of patients with complete response or partial response. The disease control rate was defined as the rate of patients with complete response, partial response or stable disease. PFS was defined as the time from the introduction of nivolumab plus ipilimumab treatment to either radiological and clinical disease progression or to death. Second progression-free survival (PFS2) was defined as the time from the introduction of nivolumab plus ipilimumab treatment to radiological and clinical disease progression on second-line treatment or death^14^. Cancer-specific survival (CSS) was defined as the time from the introduction of nivolumab plus ipilimumab treatment to death from RCC or to the patient’s last follow-up visit. OS was defined as the time from the introduction of nivolumab plus ipilimumab treatment to either death from any cause or to the patient’s last follow-up visit.

### Treatment

Patients were treated with nivolumab 3 mg/kg plus ipilimumab 1 mg/kg every three weeks for up to four cycles, then with nivolumab monotherapy, and they continued to receive the treatment until either disease progression or to the presentation of unacceptable adverse events. After the discontinuation of nivolumab plus ipilimumab, the patients were treated with subsequent drugs according to the policy of each physician.

### Statistical analysis

All statistical analyses were performed by using JMP Pro 16. Kaplan–Meier method was used to determine PFS, PFS2, CSS and OS rates. Log rank tests were performed to compare them between patients with different FAN scores. Univariable and multivariable Cox proportional regression analyses were performed to identify predictors of PFS, PFS2, CSS and OS. In addition to the FAN score, the factors considered to influence patients’ prognoses, such as age, serum CRP level, prior resection of primary site, histologic subtype, IMDC risk classification, and number of metastatic organs, were included in the multivariable analyses. *P* < 0.05 was considered to be statistically significant in all analyses.

### Ethics approval

This study was approved by the Wakayama Medical University Institutional Review Board (approval number 3470), then by the institutional review boards of the seven other participating institutions. It was conducted in accordance with the Declaration of Helsinki.

### Consent to participate

Due to the retrospective nature of this multi-center study, the need for written informed consent was waived by the Wakayama Medical University Institutional Review Board (approval number 3470).

### Human and/or animals participants

This study is a review of existing data and does not involve any studies with human participants or animals performed by other authors.

## Results

### Patient characteristics

Table 1 shows the patient characteristics based on FAN scores. Fib-4 was > 3.5 in three patients (2%), ALBI score was ≥ 2.6 in 89 patients (58%) and NLR was > 5.0 in 45 patients (29%). As a result, the pre-treatment FAN score was '0' in 56 patients (36%), ‘1’ in 60 patients (40%), ‘2’ in 37 patients (24%) and ‘3’ in one patient (1%). Among the patients with different FAN scores, there were significant differences in age (*P* = *0.01*), CRP (*P* < 0.01), prior resection of primary site (*P* < *0.01*) and IMDC risk classification (*P* < *0.01*) (FAN score 0 vs. 1 vs. ≥ 2).

**Table 1:** Patient characteristics based on FAN score.

	Overall (N = 154)	FAN score			P value
		0 (n = 56)	1 (n = 60)	≥ 2 (n = 38)	
Age (years)	69 (60–74)	65 (56–73)	70 (64–76)	68 (58–72)	0.01
Gender, n (%)					0.41
Male	126 (82)	48 (86)	46 (77)	32 (84)	
Female	28 (18)	8 (14)	14 (23)	6 (16)	
BMI (kg/m^2^)	22.2 (19.4–24.8)	22.9 (19.9–25.7)	22.0 (19.3–25.3)	21.2 (17.8–24.3)	0.05
CRP (mg/dL)	0.65 (0.14–5.01)	0.18 (0.07–0.50)	1.78 (0.19–4.88)	8.53 (2.58–12.54)	< 0.01
Prior resection of primary site, n (%)					< 0.01
Yes	86 (56)	46 (82)	27 (45)	13 (34)	
No	68 (44)	10 (18)	33 (55)	25 (66)	
Histologic subtype, n (%)					0.06
Clear cell	101 (66)	40 (71)	40 (67)	21 (55)	
Non-clear cell	36 (23)	14 (25)	14 (23)	8 (21)	
Unknown	17 (11)	2 (4)	6 (10)	9 (24)	
Sarcomatoid features, n (%)					0.75
Yes	16 (10)	7 (13)	5 (8)	4 (11)	
No	138 (90)	49 (88)	55 (92)	34 (89)	
IMDC risk classification, n (%)					< 0.01
Intermediate	95 (62)	50 (89)	35 (58)	10 (26)	
Poor	59 (38)	6 (11)	25 (42)	28 (74)	
Number of metastatic organs, n (%)					0.80
Single	80 (52)	30 (54)	32 (53)	18 (47)	
Multiple	74 (48)	26 (46)	28 (47)	20 (53)	
Metastatic sites, n (%)					
Lung	92 (60)	35 (63)	36 (60)	21 (55)	0.78
Lymph nodes	56 (36)	15 (27)	22 (37)	19 (50)	0.07
Bone	51 (33)	17 (30)	17 (28)	17 (45)	0.21
Liver	23 (15)	6 (11)	9 (15)	8 (21)	0.39
Adrenal gland	9 (6)	6 (11)	1 (2)	2 (5)	0.10
Brain	3 (2)	2 (4)	1 (2)	0 (0)	0.62
Others	32 (21)	14 (25)	12 (20)	6 (16)	0.54

Continuous variables are shown in "median (IQR)" form.

*BMI* body mass index; *CRP* C-reactive proten; *IMDC* International Metastatic RCC Database Consortium.

### Association between FAN score and treatment response

**Figure 1:**
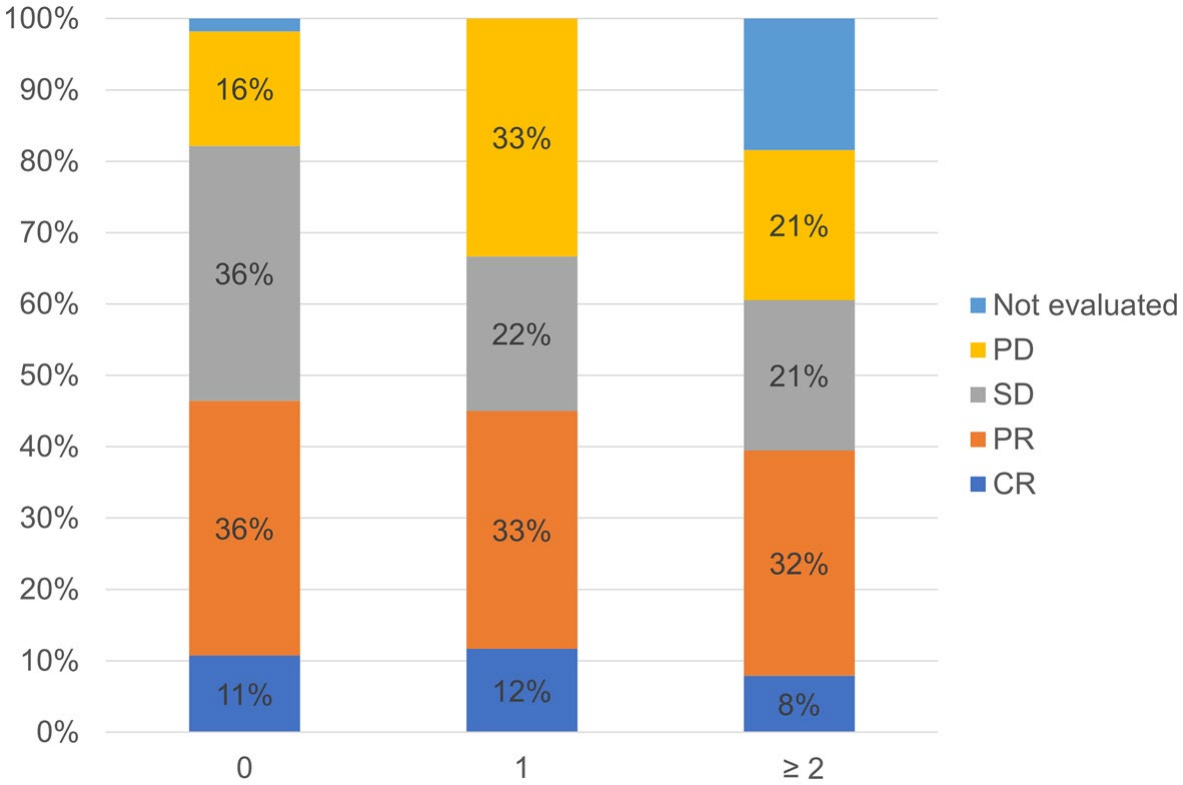
Association between pre-treatment FAN score and treatment response. CR: complete response, PR: partial response, SD: stable disease and PD: progressive disease.

The association between pre-treatment FAN score and treatment response is shown in Fig. 1. ORR was 46%, 45% and 39% in patients with FAN score 0, 1 and ≥ 2, respectively (*P* = *0.78*). Disease control rate was significantly different between the three groups: 82%, 67% and 61% in patients with FAN scores of 0, 1 and ≥ 2, respectively (*P* = *0.04*).

### Association between FAN score and survival outcomes

**Figure 2:**
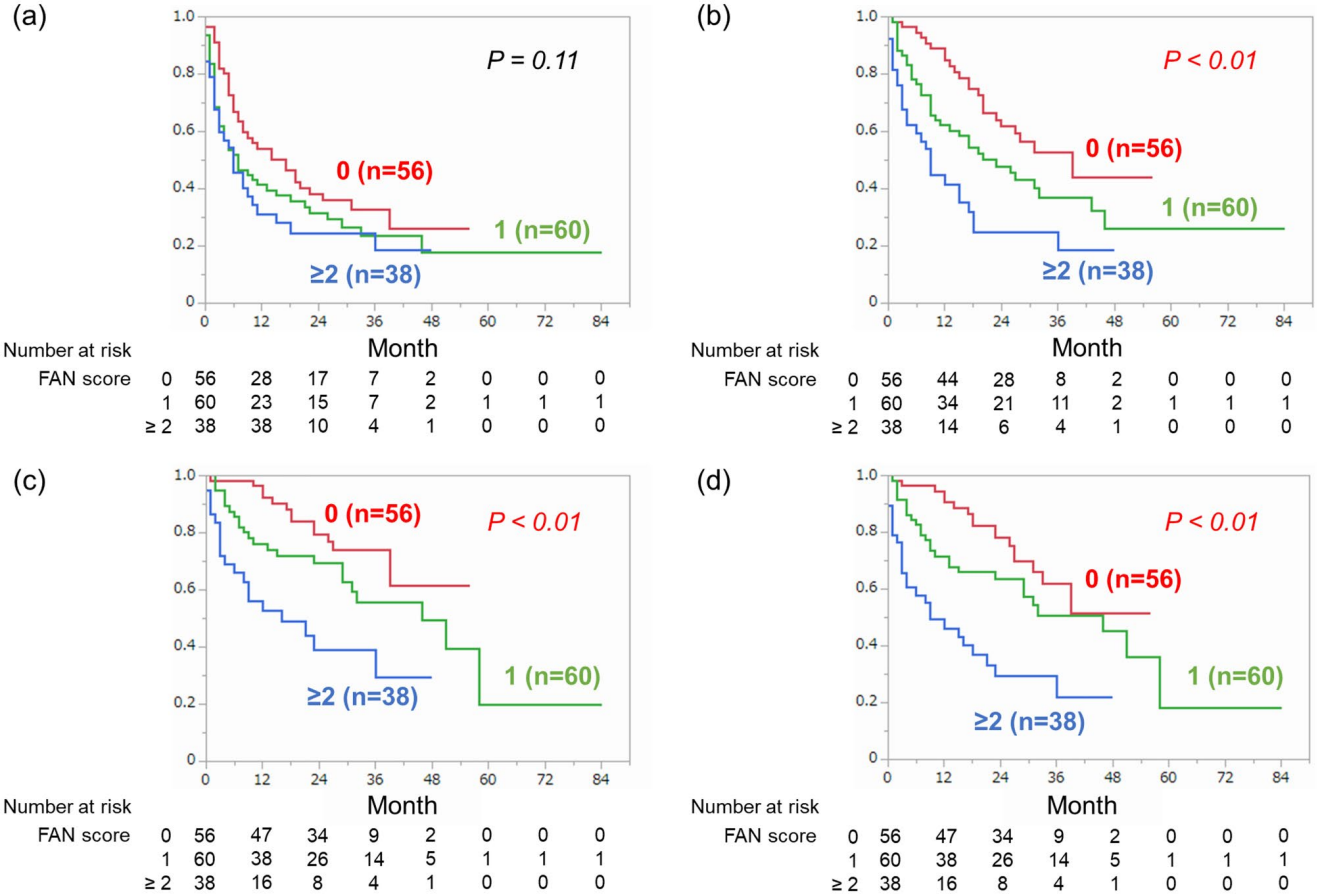
Comparison of (**a**) progression-free survival, (**b**) second progression-free survival, (**c**) cancer-specific survival and (**d**) overall survival between patients with different FAN score.

During the median follow-up period of 21 months (quartile: 8–31 months), 76 patients (50%) patients received subsequent second-line treatment after nivolumab plus ipilimumab. Fifty-six patients (37%) and 14 patients (9%) died of RCC and of other causes, respectively. As shown in Fig. 2a, there was no significant difference of PFS among patients with different FAN scores (0: median 17 [95% CI 7–25] vs. 1: 7 [3–15] vs. ≥ 2: 6 [3–10] months, *P* = *0.11*). On the other hand, PFS2 was significantly different between the groups (0: median 39 [95% CI 23-not reached] vs. 1: 23 [11–43] vs. ≥ 2: 9 [4–17] months, *P* < *0.01*) (Fig. 2b). In addition, there were also significant differences of CSS (0: median not reached [95% CI 39-not reached] vs. 1: 46 [29-not reached] vs. ≥ 2: 16 [6-not reached] months, *P* < *0.01*) and OS (0: median not reached [95% CI 31-not reached] vs. 1: 46 [23–58] vs. ≥ 2: 9 [3–21] months, *P* < *0.01*) between the groups (Fig. 2c,d).

### Predictive value of FAN score for outcomes

As shown in Table 2, while age and no prior resection of the primary site were independent predictors of poor PFS (*P* = *0.04* and *P* = *0.02*, respectively), the pre-treatment FAN score was not. On the other hand, FAN score ≥ 2 was an independent predictor of poor PFS2 (*P* = *0.01*) in addition to no prior resection of the primary site (*P* = *0.02*) and multiple metastatic organs (*P* = *0.02*), as shown in Table 3. Moreover, FAN score ≥ 2 was independently associated with poor CSS (*P* = *0.02*) and poor OS (*P* = *0.02*) as well as no prior resection of the primary site (*P* = *0.01* and *P* = *0.01*) and histologic subtype (*P* < *0.01* and *P* < *0.01*), as shown in Tables 4 and 5.

**Table 2:** Univariable and multivariable analyses of factors associated with progression free survival.

	Univariable analysis			Multivariable analysis		
	HR	95% CI	P value	HR	95% CI	P value
Age: ≥ 69 years (vs. < 69 years)	0.81	0.55–1.17	0.26	0.67	0.44–0.99	0.04
BMI: < 22.2 kg/m^2^ (vs. ≥ 22.2 kg/m^2^)	1.40	0.95–2.04	0.08			
CRP: ≥ 0.65 mg/dL (vs. < 0.65 mg/dL)	1.68	1.14–2.45	< 0.01	1.25	0.76–2.05	0.37
Prior resection of primary site: No (vs. Yes)	1.92	1.31–2.80	< 0.01	1.64	1.05–2.52	0.02
Histologic subtype: clear cell (vs. others)	0.68	0.46–1.00	0.05	0.86	0.56–1.31	0.49
Sarcmatoid features: Yes (vs. No)	0.98	0.52–1.83	0.95			
IMDC risk classification: poor (vs. intermediate)	1.32	0.90–1.94	0.15	1.02	0.62–1/64	0.95
Number of metastatic organs: multiple (vs. single)	1.41	0.96–2.05	0.07	1.26	0.85–1.86	0.24
FAN score						
0	(reference)			(reference)		
1	1.38	0.88–2.14	0.15	1.15	0.68–1.95	0.59
≥ 2	1.62	0.98–2.65	0.05	1.17	0.62–2.22	0.63

*BMI* body mass index; *CRP* C-reactive proten; *IMDC* International Metastatic RCC Database Consortium.

**Table 3:** Univariable and multivariable analyses of factors associated with progression free survival 2.

	Univariable analysis			Multivariable analysis		
	HR	95% CI	P value	HR	95% CI	P value
Age: ≥ 69 years (vs. < 69 years)	0.88	0.57–1.34	0.54	0.72	0.46–1.11	0.14
BMI: < 22.2 kg/m^2^ (vs. ≥ 22.2 kg/m^2^)	1.47	0.96–2.26	0.07			
CRP: ≥ 0.65 mg/dL (vs. < 0.65 mg/dL)	2.33	1.49–3.61	< 0.01	1.28	0.71–2.28	0.40
Prior resection of primary site: No (vs. Yes)	2.44	1.58–3.76	< 0.01	1.78	1.09–2.88	0.02
Histologic subtype: clear cell (vs. others)	0.53	0.34–0.81	< 0.01	0.68	0.42–1.07	0.09
Sarcmatoid features: Yes (vs. No)	1.04	0.49–2.14	0.92			
IMDC risk classification: poor (vs. intermediate)	1.55	1.01–2.38	0.04	0.83	0.48–1.41	0.49
Number of metastatic organs: multiple (vs. single)	1.89	1.22–2.91	< 0.01	1.65	1.05–2.58	0.02
FAN score						
0	(reference)			(reference)		
1	1.70	1.00–2.88	0.04	1.41	0.76–2.59	0.27
≥ 2	3.15	1.79–5.52	< 0.01	2.43	1.21–4.87	0.01

*BMI* body mass index; *CRP* C-reactive proten; *IMDC* International Metastatic RCC Database Consortium.

**Table 4:** Univariable and multivariable analyses of factors associated with cancer-specific survival.

	Univariable analysis			Multivariable analysis		
	HR	95% CI	P value	HR	95% CI	P value
Age: ≥ 69 years (vs. < 69 years)	0.72	0.42–1.22	0.22	0.62	0.35–1.07	0.08
BMI: < 22.2 kg/m^2^ (vs. ≥ 22.2 kg/m^2^)	1.32	0.77–2.24	0.30			
CRP: ≥ 0.65 mg/dL (vs. < 0.65 mg/dL)	2.96	1.66–5.26	< 0.01	1.59	0.77–3.27	0.20
Prior resection of primary site: No (vs. Yes)	2.95	1.70–5.12	< 0.01	2.07	1.12–3.79	0.01
Histologic subtype: clear cell (vs. others)	0.32	0.18–0.54	< 0.01	0.44	0.24–0.77	< 0.01
Sarcmatoid features: Yes (vs. No)	1.27	0.54–2.97	0.58			
IMDC risk classification: poor (vs. intermediate)	1.46	0.86–2.48	0.15	0.72	0.38–1.34	0.30
Number of metastatic organs: multiple (vs. single)	1.48	0.86–2.52	0.14	1.30	0.75–2.24	0.33
FAN score						
0	(reference)			(reference)		
1	1.76	0.88–3.52	0.10	1.31	0.59–2.91	0.50
≥ 2	4.03	1.99–8.14	< 0.01	2.71	1.13–6.47	0.02

*BMI* body mass index; *CRP* C-reactive proten; *IMDC* International Metastatic RCC Database Consortium.

**Table 5:** Univariable and multivariable analyses of factors associated with overall survival.

	Univariable analysis			Multivariable analysis		
	HR	95% CI	P value	HR	95% CI	P value
Age: ≥ 69 years (vs. < 69 years)	0.90	0.56–1.43	0.65	0.78	0.48–1.27	0.32
BMI: < 22.2 kg/m^2^ (vs. ≥ 22.2 kg/m^2^)	1.54	0.95–2.47	0.07			
CRP: ≥ 0.65 mg/dL (vs. < 0.65 mg/dL)	3.02	1.80–5.06	< 0.01	1.68	0.87–3.20	0.11
Prior resection of primary site: No (vs. Yes)	2.76	1.69–4.50	< 0.01	1.93	1.12–3.30	0.01
Histologic subtype: clear cell (vs. others)	0.37	0.23–0.60	< 0.01	0.50	0.30–0.83	< 0.01
Sarcmatoid features: Yes (vs. No)	1.16	0.52–2.53	0.71			
IMDC risk classification: poor (vs. intermediate)	1.62	1.01–2.59	0.04	0.78	0.44–1.39	0.41
Number of metastatic organs: multiple (vs. single)	1.60	0.99–2.57	0.05	1.37	0.84–2.22	0.20
FAN score						
0	(reference)			(reference)		
1	1.60	0.86–2.96	0.13	1.12	0.55–2.27	0.75
≥ 2	3.94	2.12–7.29	< 0.01	2.42	1.11–5.25	0.02

*BMI* body mass index; *CRP* C-reactive proten; *IMDC* International Metastatic RCC Database Consortium.

## Discussion

The potential for FAN score to be used as a prognostic marker in patients with urothelial carcinoma receiving pembrolizumab has been previously reported, but there has not been a proper evaluation of the usefulness of FAN score for predicting prognostic outcomes after ICI treatment in metastatic RCC. To the best of our knowledge, this is the first report on the prognostic impact of-pre-treatment FAN score in patients with RCC receiving nivolumab plus ipilimumab. In this multi-center retrospective study, we examined the association between pre-treatment FAN score and prognostic outcomes in patients treated with nivolumab plus ipilimumab treatment for metastatic RCC. Patients with high pre-treatment FAN scores had lower rates of PFS2, CSS and OS compared with those with low FAN scores. Moreover, FAN score ≥ 2 was a significant independent predictor of poor PFS2, CSS and OS.

The original results of the Checkmate 214 trial showed that nivolumab plus ipilimumab had clinical benefits for patients with untreated IMDC intermediate/poor risk RCC compared with sunitinib^2^. Extended follow-up results of the trial also demonstrated that this combination therapy had durable clinical efficacy^9^. Therefore, while various novel ICI regimens have been approved, nivolumab plus ipilimumab is still recommended as the first-line treatment regimen for patients with IMDC intermediate/poor risk RCC^8^. The IMDC risk classification has been proposed as a prognostic classification for patients receiving molecular targeted drugs and has been widely used in clinical practice, but a prognostic classification for patients receiving ICIs including nivolumab plus ipilimumab has not yet been well established^15^. To select patients suitable for nivolumab plus ipilimumab for metastatic RCC, development of novel prognostic biomarkers or nomograms which may be easily used in daily practice is highly anticipated.

The FAN score, a focus of the present study, is a novel prognostic scoring system for patients treated with ICIs and based on Fib-4 index, ALBI score and NLR. The Fib-4 index was originally proposed as an indicator of liver fibrosis in patients with viral hepatitis^11^. The cutoff value of 3.5 for Fib-4 index is used as an indicator of liver cirrhosis and is also used to calculate the FAN score^10,16^. ALBI score was originally proposed as a novel indicator of functional liver reserve for patients with hepatocellular carcinoma^17^. The cutoff value of -2.6 for ALBI score is widely accepted, and is also used in the calculation of the FAN score^10,17^. These scoring systems are not just indicators of liver function, they are reportedly associated with prognosis in patients with hepatocellular carcinoma treated with ICIs, cardiac disease and coronavirus disease^18–24^. There is reported association between ALBI score and prognosis of patients with various cancers other than hepatocellular carcinoma^25,26^. In addition, pre-treatment ALBI grade was recently reported to be associated with prognosis in patients with lung cancer or urothelial carcinoma treated with ICIs^12,27,28^. The liver is an important organ in relation to protein production and regulation of innate and acquired immunity^29^. Liver dysfunction could affect the interaction between various cytokines and lead to changes of T cell subset repertoires, which might explain why these scoring systems are associated with the prognosis of patients with cancer that are treated with ICIs^30,31^. In addition, these scoring systems could reflect general conditions related to cachexia^10^. Cancer-related cachexia is induced by changes of systemic metabolic environment due to elevation of inflammatory cytokines in tumor cells, hepatocytes and adipose tissue, changes of protein synthesis and degradation in skeletal muscle and insulin resistance. It leads to weight loss, anorexia and decreased systemic function^32–39^.

NLR, meanwhile, is widely known to be an indicator of systematic inflammatory responses. The elevation of neutrophil counts induces the secretion of cytokines, such as interleukin-1β and interleukin-12. Tumor necrosis factor-α promotes a chronic inflammation condition and inhibits natural killer cells and effector T-cell-mediated antitumor immunity^40,41^. The prognostic role of NLR in urological cancers including RCC has not been widely reported^42^. Meanwhile, there have been reports of the prognostic role of NLR in patients with metastatic RCC treated with nivolumab plus ipilimumab^43,44^. Our results, that FAN score had a significant impact on the prognosis of metastatic RCC patients receiving nivolumab plus ipilimumab, are thus thought to be reasonable.

Kawashima et al. established FAN score by using the data of 165 patients with metastatic urothelial carcinoma receiving pembrolizumab as a validation cohort^10^. FAN score was significantly associated with both PFS and CSS. Moreover, they also examined the association between FAN score and prognosis in a validation cohort including 103 patients with metastatic urothelial carcinoma treated with pembrolizumab. FAN score was also a significant predictor of PFS and CSS. In our study, pre-treatment FAN score was not significantly associated with PFS, but FAN score ≥ 2 was a significant predictor of poor PFS2 (vs. FAN score 0, HR: 2.43, 95% CI 1.21–4.87, *P* = *0.01*), poor CSS (vs. FAN score 0, HR: 2.71, 95% CI 1.13–6.47, *P* = *0.02*) and poor OS (vs. FAN score 0, HR: 2.42, 95% CI 1.11–5.25, *P* = *0.02*). PFS might not be a surrogate for OS in patients receiving ICI, but ICI could provide delayed benefit^45^, and our results might align with this. Studies on the association between FAN score and prognosis in patients with malignancies receiving ICIs are still limited, so further validation of the prognostic value of FAN score is required.

We found no prior resection of the primary site to also be significantly associated with poor prognostic outcomes. Subgroup analyses of the Checkmate 214 trial reported that nivolumab plus ipilimumab provided survival benefits and volume reduction of the primary site in patients without prior nephrectomy^46^. However, Kato et al. analyzed the real-world data of 72 Japanese patients with metastatic RCC and receiving nivolumab plus ipilimumab; they reported that median OS in patients with prior nephrectomy was better than in those without (not reached vs. 25.3 months, *P* = 0.028) and that no prior nephrectomy was an independent predictor of poor OS^47^. Stellato et al. also retrospectively evaluated the real-world data of patients with metastatic RCC receiving ICIs. Median OS in patients who underwent previous nephrectomy was better than in those who did not (20.9 months vs. 13 months, *P* = 0.001) and prior nephrectomy was an independent predictor of better outcome in terms of both OS and PFS^48^. Our results were consistent with the results of these previous studies.

The present study has several limitations. First, it is a retrospective study with a limited number of patients, which could lead to a selection bias. However, the number of patients in this study is comparable with that in previous studies which reported the clinical outcomes of nivolumab plus ipilimumab for metastatic RCC in real-world clinical practice. Second, the follow-up duration after the introduction of nivolumab plus ipilimumab was relatively short (median: 21 months). However, the prognosis of patients with IMDC intermediate/poor risk is generally poor and 70 patients (45%) from our cohort died (all causes) during the follow-up duration. Third, although we carefully selected and investigated the patient background and tumor characteristic factors to be collected, there might still be confounding factors which could not be assessed in the present study. Further large-scale studies will seek to verify our results.

## Conclusion

In conclusion, high pre-treatment FAN score could be associated with poor prognosis in patients treated with nivolumab plus ipilimumab. Pre-treatment evaluation of FAN score may be clinically useful for predicting prognosis in patients receiving nivolumab plus ipilimumab treatment.

## Data Availability

The datasets analyzed during the current study are available from the corresponding author on reasonable request.

## Abbreviations

ALBI: Albumin–bilirubin
ALT: Alanine aminotransferase
AST: Aspartate aminotransferase
CRP: C-reactive protein
CSS: Cancer-specific survival
DCR: Disease control rate
Fib-4: Fibrosis-4
ICIs: Immune checkpoint inhibitors
IMDC: International Metastatic Renal Cell Carcinoma Database Consortium
NLR: Neutrophil–lymphocyte ratio
ORR: Objective response rate
OS: Overall survival
PD-1: Programmed death-1
PFS2: Second progression-free survival
RCC: Renal cell carcinoma
